# Advancing neoadjuvant therapies in resectable non-small cell lung cancer: implications for novel treatment strategies and biomarker discovery

**DOI:** 10.3389/pore.2024.1611817

**Published:** 2024-06-18

**Authors:** Hyein Jeon, Rajvi Gor, Angelica D’Aiello, Brendon Stiles, Peter B. Illei, Balazs Halmos

**Affiliations:** ^1^Department of Oncology, Montefiore Medical Center, Albert Einstein College of Medicine, Bronx, NY, United States; ^2^ Department of Medicine, Jacobi Medical Center, Bronx, NY, United States; ^3^Department of Cardiothoracic and Vascular Surgery, Montefiore Medical Center, Albert Einstein College of Medicine, Bronx, NY, United States; ^4^Department of Pathology, The Johns Hopkins Hospital, Johns Hopkins Medicine, Baltimore, MD, United States

**Keywords:** NSCLC, perioperative, neoadjuvant, adjuvant, lung cancer

## Abstract

The delivery of neoadjuvant and perioperative therapies for non-small cell lung cancer has been radically altered by significant advances and by the incorporation of targeted therapies as well as immune checkpoint inhibitors alone or alongside conventional chemotherapy. This evolution has been particularly notable in the incorporation of immunotherapy and targeted therapy into the treatment of resectable NSCLC, where recent FDA approvals of drugs such as nivolumab and pembrolizumab, in combination with platinum doublet chemotherapy, have led to considerable improvements in pathological complete response rates and the potential for enhanced long-term survival outcomes. This review emphasizes the growing importance of biomarkers in optimizing treatment selection and explores the impact of emerging studies that challenge existing treatment paradigms and investigate novel therapeutic combinations poised to redefine standard of care practices. Furthermore, the discussion extends to the unmet needs within perioperative treatment assessment and prognostication, highlighting the prospective value of biomarkers in evaluating treatment responses and prognosis.

## Introduction

Lung cancer, with non-small cell lung cancer (NSCLC) representing about 80% of cases, continues to pose a formidable health issue, ranking as the second highest in new cancer cases and the leading cause of cancer mortality worldwide [1]. The landscape of NSCLC management has undergone dramatic changes in recent years, driven by the advent of biomarker-targeted therapies and immunotherapies. These advances have not only transformed the treatment of advanced and locally advanced disease but are now rapidly reshaping the approach to resectable NSCLC as well. In the perioperative setting for resectable NSCLC, nivolumab and more recently pembrolizumab with platinum doublet chemotherapy have been approved in neoadjuvant/perioperative settings (Table 1) and pembrolizumab, atezolizumab, osimertinib, and alectinib all approved in the adjuvant setting, respectively. These approvals in the perioperative settings have markedly improved the management of resectable NSCLC, heralding a new era where molecularly targeted therapies and immune checkpoint inhibitors are poised to optimize treatment efficacy. This transformative phase is set against a contrasting historical context of two decades marked by numerous attempts to augment the standard of adjuvant chemotherapy, most of which failed to improve outcomes significantly. High-profile endeavors like the integration of radiation therapy, angiogenesis inhibition through VEGF targeting [2], and cancer vaccines targeting specific antigens such as MAGE [3] have been rigorously investigated but ultimately did not achieve a new standard of care, reflecting the complexity and resilience of NSCLC to therapeutic advances. This review seeks to provide a comprehensive overview of the current state and future directions of perioperative treatment in NSCLC, highlighting biomarker identification that could refine treatment selection and improve clinical outcomes, as well as exploring novel therapeutics to redefine the standards of care for NSCLC.

**TABLE 1:** Key phase III studies for preoperative regimens.

Trial	Regimen and FDA approval	Stage	ALK/EGFR included	Patients	PFS/EFS/RFS/OS	pCR/mPR
CheckMate 816 (8)	Neoadjuvant **nivolumab** + CT vs. CT for 3 cycles **Approved**	IB-IIIA (AJCC 7th ed)	No	358	**EFS** at 2 years 63.8% vs. 45.3% (**HR** 0.65 CI 0.43–0.91) **mEFS** 31.6 vs. 20.8 months (HR 0.63 CI 0.43–0.91 *p* = 0.005) **OS** at 2 years not reached in either arms (HR 0.57 CI 0.30–1.07 *p* = 0.008)	**pCR** 24% vs. 2.2% (OR 13.94 CI 18.0–31.0 *p* < 0.001) **mPR** 36.9% vs. 8.9% (OR 5.7 CI 3.16–10.26)
NADIMII (12)	Neoadjuvant **nivolumab** + CT vs. CT for 3 cycles R0 resections → adjuvant **nivolumab** for 6 months	IIIA-IIIB	No	86	**PFS** at 2 years 67.2% vs. 40.9% (HR 0.47 CI 0.25–0.88) **OS** at 2 years 85% vs. 63.6% (HR 0.43 CI 0.19–0.98)	**pCR** 37% vs. 7% (RR 5.34 CI 1.34–21.23 *p* = 0.02) **mPR** 53% vs. 14% (RR 3.82 CI 1.49–9.79)
KEYNOTE-671 (9)	Neoadjuvant **pembrolizumab** + CT for 4 cycles vs. placebo + CT Adjuvant **pembrolizumab** monotherapy up to 13 cycles vs. placebo **Approved**	II-IIIB N2 (AJCC 8th ed)	Yes	797	**EFS** at 2 years 62.4% vs. 40.6% (HR 0.58 CI 0.46–0.72 *p* < 0.001) **OS** at 2 years 80.9% vs. 77.6% **Median OS** NR vs. 45.5 months (*p* = 0.02)	**pCR** 18.1% vs. 4.0% (difference 14.2% CI 10.1–18.7 *p* < 0.0001) **mPR** 30.2% vs. 11.0% (difference 19.2% CI 13.9–24.7 *p* < 0.0001)
AEGEAN (17)	Neoadjuvant **durvalumab** + CT for 4 cycles vs. placebo + CT Adjuvant **durvalumab** monotherapy up to 12 cycles vs. placebo	II-IIIB N2 (AJCC 8th ed)	No	740	**EFS** at 2 years 63.3% vs. 52.4% (HR 0.68 CI 0.53–0.88 *p* = 0.04)	**pCR** 17.2% vs. 4.3% **mPR** 33.3% vs. 12.3%
CheckMate 77T (20)	Neoadjuvant **nivolumab** + CT for 4 cycles vs. placebo + CT Adjuvant **nivolumab** vs. placebo for 1 year	IIA-IIIB (N2)	No	461	**mEFS** not reached vs. 18.4 months (HR 0.58 CI 0.42–0.81 *p* = 0.00025)	**pCR** 25.3% vs. 4.3% (OR 6.64 CI 3.4–12.97) **mPR**35.4% vs. 12.1% (OR 4.01 CI 2.48–6.49)

CT, chemotherapy; DFS, disease-free survival; EFS, event-free survival; HR, hazard ratio; mEFS, median event-free survival; OR, odds ratio; OS, overall survival; PFS, progression free survival; R0, complete resection; RFS, recurrence-free survival; RR, relative risk.

### Current FDA approved preoperative standard of care for resectable NSCLC

The current standard of care for the neoadjuvant treatment of resectable NSCLC has evolved substantially, now increasingly utilizing multimodal strategies to improve surgical outcomes and hopefully to extend overall survival (OS). While studies in the past have indicated similar benefits between neoadjuvant and adjuvant chemotherapy [4, 5], logistical considerations sustained the latter as the prevailing practice pattern. Nonetheless, for patients with stage IIA to IIIA resectable NSCLC, the standard neoadjuvant protocol has conventionally incorporated platinum-based doublet chemotherapy, which has proven to enhance survival rates when compared with surgery alone [6].

Recent developments have also introduced immune checkpoint inhibitors into the neoadjuvant setting for NSCLC, either as monotherapy, concomitantly with chemotherapy, or in the context of dual immunotherapeutic strategies (Table 1). The unique aspect of the neoadjuvant approach is that it provides clinicians with the opportunity to directly observe the patient’s tumor response to treatment through the assessment of the postsurgical specimen. This pathological assessment can offer invaluable insights into the efficacy of the neoadjuvant regimen and the tumor’s biological behavior under therapeutic pressure. In addition, the native tumor serving as an *in situ* “tumor vaccine” might provide optimal T cell responses as opposed to administration post-operatively in a micrometastatic setting. Monotherapy with immune checkpoint inhibitors has allowed for novel translational studies, but have yielded moderate efficacy [7] while combination chemotherapy and immunotherapy has shown more promising results from clinical trials, specifically significant improvements in pathological complete response rates and potentially long-term outcomes [8, 9]. Recent investigations predominantly gravitate towards the synergistic potential of perioperative chemotherapy combined with immune checkpoint inhibitors, underscored by clinical trial evidence suggesting substantial improvement in rates of pathological complete response, with the prospective to confer sustained survival benefits.

### Current neoadjuvant immunotherapy studies

Nivolumab in combination with platinum doublet chemotherapy (PDC) was approved in the neoadjuvant setting for resectable NSCLC (Stage IIA to IIIA per AJCC eighth edition) without known driver mutations. The approval was based on the pioneering CheckMate 816 study which demonstrated a significantly improved EFS at 2 years of 63.8% versus 45.3% and HR of 0.65 (95% CI 0.47–0.90) of neoadjuvant chemo/immunotherapy versus PDC alone. Median EFS was 31.6 (95% CI 30.2-not reached) vs. 20.8 months (95% CI 14–26.7). The pathological complete response (pCR) after neoadjuvant chemoimmunotherapy (3 cycles) was also notably higher at 24% compared to 2.2% with chemotherapy alone demonstrating a dramatic effect on improving tumor response [8].

Several perioperative studies involving both use of neoadjuvant and adjuvant immunotherapy offer further new insights into management and treatment in the perioperative setting. The NADIM trial utilized the anti-PD1 agent, nivolumab in the perioperative setting along with PDC (carboplatin/paclitaxel) in the neoadjuvant setting followed by adjuvant nivolumab monotherapy for 1 year in 46 patients with stage IIIA NSCLC and showed 83% major pathological response (mPR), 63% pCR, with OS of 81.9% at 36 months [10, 11]. In the subsequent NADIM II trial, cohorts were expanded to stage IIIA and IIIB disease comparing chemoimmunotherapy versus PDC alone in the neoadjuvant setting followed by adjuvant nivolumab post-surgery for 6 months in those who underwent R0 resections and received nivolumab preoperatively. This trial has similarly demonstrated impressive findings [12], further supporting the argument for the use of perioperative use of immunotherapy in resectable NSCLC (see Table 1).

The recent FDA approval for perioperative use of pembrolizumab was based on results of the pivotal KEYNOTE-671 study where pembrolizumab along with PDC significantly improved pathological response as well as event free (EFS) and overall survival (OS). KEYNOTE-671 using neoadjuvant (4 cycles) and adjuvant (up to 13 cycles) pembrolizumab and platinum-based chemotherapy in stage II-IIIB (N2 stage) also showed a significant EFS HR of 0.58 with 62.4% versus 40.6% EFS at 24 months in the experimental versus the control arm of neoadjuvant PDC alone. Additionally, mPR was 30.2% versus 11.0%, pCR 18.1% versus 4%, and OS at 2 years of 80.9% versus 77.6% in the pembrolizumab compared to placebo group respectively. Interestingly, exploratory analysis has shown potential benefits in the perioperative use of pembrolizumab in those without mPR or pCR as well (HR 0.73 and 0.69 respectively) [9]. Also notably, the KEYNOTE-671 study included some patients with Epidermal Growth Factor Receptor (EGFR) and Anaplastic Lymphoma Kinase (ALK) mutations—subgroups historically with limited benefit from immunotherapy [13–16].

Other perioperative studies such as AEGEAN and NEOTORCH have demonstrated similar benefits. The randomized AEGEAN trial which studied durvalumab versus placebo along with PDC in the neoadjuvant setting (4 cycles) followed by adjuvant durvalumab or placebo monotherapy up to 12 cycles in stage II-IIIB (N2 stage) NSCLC showed EFS at 24 months of 63.3% compared to 52.4% with HR of 0.68 with median EFS not met in durvalumab group versus 25.9 months in the placebo group. mPR was 33.3% versus 12.3% while pCR was 17.2% versus 4.3% [17]. Toripalimab (3 cycles in neoadjuvant and 1 cycle in adjuvant setting with PDC followed by toripalimab alone up to 13 cycles) was also administered to stage II-IIIB (N2 stage) NSCLC without EGFR or ALK mutations in NEOTORCH trial in China which also showed significant benefits with EFS at 2 years was 64.7% versus 38.7% with HR of 0.4 with median EFS not reached in toripalimab versus 15.1 months in the placebo arm with mPR of 48.5% versus 8.4% and pCR of 24.8% versus 1.0% [18].

Dual neoadjuvant immunotherapy with nivolumab and ipilimumab was also studied in the phase II NEOSTAR trial. Combination of ipilimumab and nivolumab compared to nivolumab alone improved mPR rate to 50% vs. 24% respectively [19]. The addition of chemotherapy to neoadjuvant ipilimumab plus nivolumab resulted in a mPR rate of 50%, compared to 32% with nivolumab alone [20].

### Emerging perioperative studies

Unresolved challenges in the management of perioperative NSCLC include a need for deeper understanding of patient selection and better methods to determine treatment duration, in particular an improved prognostication of whether adjuvant therapy is needed in patients who received neoadjuvant chemoimmunotherapy. The design of the CheckMate 816 trial stands out for its focused examination of neoadjuvant treatment, demonstrating the clear benefits of incorporating neoadjuvant immunotherapy. This approach contrasts with other perioperative studies that combine both neoadjuvant and adjuvant treatments, a methodological choice that cannot separate the contribution of each component of therapy. The targeted approach used in CheckMate 816 trial was endorsed as the preferred design schematic by the FDA [21], setting a new standard for the design of perioperative clinical trials in this domain. Preliminary interim analysis from CheckMate 77T which compares neoadjuvant nivolumab with PDC and adjuvant nivolumab for 1 year to neoadjuvant placebo with PDC and adjuvant placebo for 1 year has met its primary endpoint of EFS (not reached vs. 18.4 months, HR 0.58), pCR of 25.3% vs. 4.7%, mPR of 35.4% vs. 12.1% with comparable tolerability as of now and awaits data maturation [22]. As such, many additional neoadjuvant immunotherapy (NCT06269211 (toripalimab)) and chemoimmunotherapy trials (NCT05962021 (toripalimab+PDC), NCT05157776 (sintilimab+PDC), NCT05882513 (serplulimab+PDC), NCT06241807 (camrelizumab+PDC)) are underway as well as perioperative studies (NCT05925530 (durvalumab+PDC followed by surgery and adjuvant durvalumab vs. chemoradiotherapy), NCT05116462 (sintilimab + PDC pre and post operatively followed by maintenance sintilimab vs. placebo). The results of these studies are awaited to further define the best use of perioperative therapy. However, none of them have a design that will allow clear understanding of the added value of the adjuvant treatment component.

There is consequently a pressing need for expanded research focused on patients who do not achieve a pCR following neoadjuvant therapy and for new studies incorporating novel biomarkers and experimental strategies. Additionally, despite the quite excellent outcomes of this group of patients, the role of continuing immunotherapy post-operatively in patients who have achieved pCR remains an open question. Refined biomarkers and prognostic tools are essential for precisely selecting patients likely to derive maximal benefit from adjuvant immunotherapy post-curative resection, thereby minimizing cumulative toxicities, alleviating treatment-related burdens, and reducing financial toxicity.

### Perioperative treatment with EGFR/ALK mutations

Concerted efforts are similarly underway to advance therapies targeting driver mutations in earlier settings. Postoperative use of osimertinib for 3 years with or without adjuvant chemotherapy, as evidenced by the pivotal ADAURA trial in patients with resected stage IB-IIIA NSCLC who have EGFR exon 19 deletions or exon 21 L858R mutationshas significantly extended both DFS (90% vs. 44% at 2 years, 73% vs. 38% at 4 years) with HR of 0.17 at 2 years and 0.23 at 4 years as well as OS and furthermore CNS disease free rate was also significantly improved- 92% vs. 81% compared to placebo [19, 20]. Alectinib use of up to 2 years in adjuvant setting, evaluated in the ALINA study for stage IB-IIIA NSCLC patients with ALK rearrangements, was compared against adjuvant platinum based chemotherapy for 4 cycles and has also shown a considerable prolongation in DFS of 93.6% vs. 63.7% at 2 years with HR 0.24 [23]. Its assessment has led to recent FDA approval of alectinib in ALK-positive NSCLC in the adjuvant setting.

Some immunotherapy-focused studies, such as KEYNOTE-671 allowed patients harboring mutations in EGFR and ALK to participate and some subset analyses based on small numbers of patients are suggestive of potential benefits. However, given the outstanding activity of targeted agents for EGFR and ALK mutation harboring patients, the focus at present should revolve around optimal perioperative utilization of targeted therapies. Indeed, several other early studies also demonstrated potential use of targeted therapies in preoperative studies. NCT03433469 using 2 cycles of neoadjuvant osimertinib in stage IA-IIIA NSCLC with EGFR mutations demonstrated 15% mPR and 44% achieved lymph node downstaging [24] as well as NCT04201756 which utilized 2 to 4 cycles of neoadjuvant afatinib, achieved 9.1% mPR and 57.6% had pathological downstaging for stage III NSCLC [25]. Combinatory studies using targeted therapies with or without chemotherapy are also being explored: NCT04351555 (NeoADAURA; phase III osimertinib vs. osimertinib plus PDC vs. placebo plus PDC neoadjuvantly followed by physician’s choice adjuvant treatment of targeted therapy with or without chemotherapy) [26], NCT04302025 (NAUTIKA1; single arm phase II neoadjuvant use of alectinib for 8 weeks) [27], and NCT05015010 (ALNEO; single arm phase II neoadjuvant use of alectinib for 2 cycles followed by adjuvant use up to 24 cycles) [28].

### Unmet needs in preoperative settings

One of the unmet needs in the preoperative setting for NSCLC is for accurate assessment of pathological response, which is integral to formulating decisions regarding postoperative therapy. Currently, surrogate endpoints such as pCR and mPR have been utilized to predict EFS and even more importantly OS. Although achieving mPR was observed to significantly correlate with improved survival in neoadjuvant chemotherapy trials [29, 30], further studies were needed to validate this in the era of immunotherapies and other therapeutics in resectable NSCLC. Recent meta-analysis of seven neoadjuvant randomized controlled trials showed that while pCR results were strongly correlative (*R* ^2^ = 0.82, β = 0.96) with EFS at 2 years, but that OS was only moderately correlative (*R* ^2^ = 0.55, β = 0.26). In addition, the association between hazard ratio of OS and EFS was poorly correlative (*R* ^2^ = 0.27, β = 0.11). This suggests that pCR, despite its strong linkage with EFS, may not be a completely accurate surrogate for the full clinical picture in assessing the long-term outcomes of neoadjuvant treatments [31]. Furthermore, given the potential for interobserver discrepancies due to the nature of estimating 0 or 10% residual tumor and non-standardized guidelines across trials and centers, the International Association for the Study of Lung Cancer (IASLC) published a guideline for pathologic assessment in neoadjuvant studies for NSCLC in 2020 to increase tumor sampling and assessment for tumors greater than 3 cm as well as inspection of the entire specimen for samples less than 3 cm in size [32, 33]. However, the impact of this guideline on clinical practice and patient outcomes remains uncertain, as its adoption and effectiveness in enhancing the precision of pathological assessments have yet to be thoroughly evaluated in diverse clinical settings. Developing and implementing universal pathological response assessment to facilitate more precise and informed clinical decisions that is timely, accurate, and reproducible in early-stage NSCLC is a critical and urgent need.

Lastly, there is a pressing need for novel treatments and innovative trial designs in the perioperative NSCLC landscape. While recent advances have introduced more effective treatment options, there remains a vast potential for discovering and integrating new therapies that could further enhance patient outcomes. The synergistic application of radiotherapy and immunotherapy has been observed to enhance immune priming, potentially contributing to new treatment avenues. Preclinical and clinical studies (in metastatic or recurrent settings) have shown that co-administration of immune check point inhibitor and radiation therapy may amplify release of major histocompatibility complex-1, tumor specific T cell response, as well as generating immune memory cells in tumor draining lymph nodes and potentially offer added clinical benefit [34–37]. Findings from a phase II clinical trial revealed that combination of neoadjuvant durvalumab with immunomodulatory doses of stereotactic radiation resulted in a higher mPR of 53.3% vs. 6.7% and although not statistically significant, three-year DFS rate of 83% compared to 69%, underscoring the potential of these combinatory approaches in improving patient outcomes [38]. Notably, however, the PACIFIC-2 trial with concurrent durvalumab and chemoradiotherapy compared to chemoradiotherapy for the treatment of unresectable stage III NSCLC did not meet its primary end point of PFS [39]. Similarly, JAVELIN trial in locally advanced head and neck cancer, the addition of avelumab to chemoradiotherapy also did not meet its primary end point of PFS [40]. A potential hypothesis behind several of these failures could be due to changes in tumor specific T cells after radiotherapy that negatively impacts the effect of immunotherapy. Select studies currently investigating the combination of radiation therapy and various immunotherapy include NCT05500092 (neoadjuvant nivolumab and chemotherapy with or without radiation), NCT04245514 (SAKK 16/18 chemotherapy followed by durvalumab followed by various radiation regimens then adjuvant durvalumab), NCT05798845 (neoadjuvant toripalimab plus radiotherapy), and NCT04933903 (NEO Rad neoadjuvant nivolumab, ipilimumab, and radiation).

Mechanistically novel therapeutics are being explored in the metastatic setting that potentially offer opportunities for patients with resectable NSCLC. Combination of different immune checkpoint inhibitors are under evaluation including a series of studies focused on T cell immunoreceptor with Ig and ITIM (TIGIT) antibody and lymphocyte-activation gene 3 (LAG-3) antibody and other novel checkpoints in a multidrug platform such as NEOCOAST which combines durvalumab with oleclumab (anti-CD73 monoclonal antibody (mAb)), monalizumab (anti-NKG2A mAb), or danvatirsen (anti-STAT3 antisense oligonucleotide) [41] (see Table 2). A phase II trial using combination of neoadjuvant nivolumab with or without relatlimab, a LAG-3 inhibitor, in stage IB-IIIA NSCLC was able to demonstrate mPR of 27% vs. 30%, DFS at 12 months of 89% vs. 93%, and OS at 12 months of 93% vs. 100% demonstrating potential for novel combinatory regimens [42]. Notably, antibody drug conjugates (ADC) targeting trophoblast cell surface antigen 2 (Trop-2), a transmembrane glycoprotein prevalent in NSCLC, are also gaining traction [43]. Sacituzumab govitecan, a Trop-2 targeted ADC, currently approved for metastatic breast and urothelial cancer based on improved PFS and OS [44–46], is now being studied in a range of lung cancer-focused studies. Furthermore, TROPION-Lung-02 phase 1b study demonstrated promising results with datopotamab deruxtecan (Dato-DXd), another Trop-2 ADC, in combination with pembrolizumab with or without chemotherapy with objective response rate of 60% (with chemotherapy) and 55% (without chemotherapy) suggesting a potential synergistic effect that enhances antitumor immunity [47]. Building on these findings, the TROPION-Lung-01 study compared Dato-DXd with docetaxel in advanced and metastatic NSCLC, revealing a median PFS of 5.6 versus 3.7 months. Significantly, the HR was 0.63 in non-squamous histology types, suggesting that newer therapeutics like Dato-DXd could be promising agents for further study in earlier stages of NSCLC [48]. Results from further studies like TROPION-Lung07 and TROPION-Lung08 are awaited to confirm its efficacy in advanced NSCLC [49, 50]. Concurrently, innovative trials are incorporating these therapeutics into early-stage treatment. For instance, the NeoCOAST-2 trial (NCT05061550) evaluates a multidrug platform including neoadjuvant Dato-DXd, durvalumab, and platinum, while NCT06055465 explores the combination of neoadjuvant sacituzumab govitecan and pembrolizumab.

**TABLE 2:** Ongoing representative perioperative studies with novel immune checkpoint inhibitors and biomarkers.

Upcoming perioperative chemoimmunotherapy trials					
Trial	Phase	Stage	Neoadjuvant treatment arm(s)	Adjuvant treatment arm(s)	Primary endpoint(s)
NCT04316364	III	II-IIIB	Adebrelimab + PDC	Adebrelimab	mPR, EFS
NCT05116462	III	II, IIIA or IIIB (resectable N2 only)	Sintilimab + PDC	Sintilimab + PDC, followed by Sintilimab	EFS
NCT04158440	III	II-IIIB (N2 only)	Toripalimab + PDC	Toripalimab vs. placebo	mPR, EFS
NCT04606303	II	IIB-IIIB without driver mutations	Toripalimab + PDC	NA	mPR
NCT05882513	II	IIA-IIIB (no N3 patient)	Serplulimab + PDC	NA	pCR
NCT05157776	III	IIIA	Sintilimab + PDC	NA	pCR
NCT04865250 (iREP)	II	II, IIIA or select IIIB (T3N2 only)	Atezolizumab + PDC	NA	mPR

Emerging novel combination trials					
Trial	Phase	Stage	Neoadjuvant treatment arm(s)	Adjuvant treatment arm(s)	Primary endpoint(s)
NCT04832854 (SKYSCRAPER-05)	II	II, IIIA, or select IIIB (T3N2 only)	Arm 1 (high PD-L1 expression): Atezolizumab + Tiragolumab. Arm 2 (Any PD-L1 expression): Atezolizumab + Tiragolumab + PDC	Arm 1 (high PD-L1 expression): Atezolizumab + Tiragolumab or PDC Arm 2 (Any PD-L1 expression): Atezolizumab + Tiragolumab	mPR, surgical delays, operative and post-operative complications, surgical cancellations related to study treatment, AE
NCT05061550 (NeoCOAST-2)	II	IIA-IIIB	Arm 1: Oleclumab + durvalumab + PDC	Arm 1: Oleclumab + durvalumab	pCR, AE
			Arm 2: Monalizumab + durvalumab + PDC	Arm 2: Monalizumab + durvalumab	
			Arm 3: Volrustomig (Dose Exploration) + PDC	Arm 3: Volrustomig	
			Arm 4: Dato-DXd + durvalumab + single agent platinum	Arm 4: durvalumab	
			Arm 5: AZD0171 + durvalumab + PDC	Arm 5: AZD0171 + durvalumab	
NCT05360979	II	II, IIIA, IIIB (T3N2)	Envafolimab + Recombinant human endostatin + PDC	Envafolimab	mPR
NCT05891080	II	IIIB-IIIC	Arm 1: Toripalimab + JS004 + PDC Arm 2: Toripalimab + PDC	NA	pCR
NCT05387109	IV	II-IIIB (IIIB only T3N2)	Penpulimab + Anlotinib	individualized per patient	pCR
NCT05742607 (MATISSE)	II	IIA-IIIA	Durvalumab + IPH5201 + PDC	Durvalumab + IPH5201	pCR, AE
NCT04040361 (EAST ENERGY)	II	IB-IIIA	Pembrolizumab + Ramucirumab	NA	mPR
NCT06088771	I, II	T1b or more advanced (>1 cm) and resectable	Dupilumab + Cemiplimab	standard of care	DLTs, mPR
NCT05577702	II	II-IIIA	Arm 1a: Tislelizumab Arm 1b: Tislelizumab and Ociperlimab Arm 1c: Tislelizumab and LBL-007 Arm 2a: Tislelizumab + PDC Arm 2c: LBL-007 + Tislelizumab + PDC	NA	mPR
NCT06077760	III	Resected Stage II, IIIA, IIIB (N2)	-	V940 + Pembrolizumab	DFS

Emerging novel biomarker/imaging focused trials					
Trial	Phase	Stage	Neoadjuvant treatment arm(s)	Adjuvant treatment arm(s)	Primary endpoint(s)
NCT04158440	III	II-III	Toripalimab + PDC	Toripalimab	PD-L1 in tissue specimen, TMB, WES and change of ctDNA in peripheral blood sample
NCT06221462 (PRIORITY)	II	IB-IIIB	Sintilimab + Anlotinib	Sintilimab	MRD ctDNA
NCT05429463 (neoSCORE II)	III	cIB-IIIA	Sintilimab + PDC	+/− RT, ± Sintilimab	PD-L1, ctDNA, TIIC
NCT04061590	II	I-IIIA	Treatment Arm-1: Pembrolizumab Treatment Arm-2: Pembrolizumab + PDC	NA	Proportion of patients with a ≥2-fold increase in the number of TIICs in post- versus pre-pembrolizumab treatment tumor specimens
NCT04638582	II	IA3 - IIA	Arm 1: Pembrolizumab Arm 2: Pembrolizumab + PDC	Arm 1 and 2: Pembrolizumab ± PDC	ctDNA resolution, imaging measures of response in correlation with pCR
NCT05925530 (MDT-BRIDGE)	II	IIB - IIIB (N2 only)	Durvalumab or Durvalumab followed by CRT	Durvalumab up to 12 months	ctDNA clearance
NCT02818920 (TOP1501)	II	IB (>/= 3 cm per CT), cIIA/IIB, IIIA (N0-2)	Pembrolizumab	Adjuvant CT ± RT f/b Pembrolizumab x 4	change in biomarkers, TIL, T cells specific against TAA, change in immunomodulatory effects, circulating T cells, gene expression of the PD-1/PD-L1 axis, correlation of pathologic response to the presence of TILs
NCT05882513	II	IIA-IIIB (no N3 patient)	Serplulimab + PDC	NA	ORR before surgery - the proportion of subjects with imaging PR or CR
NCT04875585 (IMNWOP1)	II	IA3-IIIA (max. single station N2)	Pembrolizumab + Lenvatinib	Pembrolizumab	radiologic response, surgical resection rate
NCT04586465 (DYNAPET)	II	IIA-IIIB	Pembrolizumab + PDC	NA	mPR, dynamic PET-CT SUV change, ORR, uptake rate constant changes
NCT06241807	II	IIIA-IIIB (T3-4N2)	Camrelizumab + PDC	NA	ctDNA, serum metabolomics testing, 18F-FDG uptake value, circulating immunological biomarkers, and observation of tumor immune microenvironment

AE, adverse events; CR, complete response; CT, chemotherapy; ctDNA, circulating-tumor DNA; DFS, disease free survival; DLTs, dose-limiting toxicities; EFS, event-free survival; mPR, major pathological response; MRD, minimal residual disease; ORR, objective response rate; ORR, objective response rate; pCR, pathological complete response; PD-L1, programmed cell death ligand 1; PDC, platinum doublet chemotherapy; PET-CT SUV, positron-emission tomography and a computed tomography standardized uptake values; PR, partial response; RT, radiation therapy; SAE, serious adverse events; TAA, tumor-associated antigen; TIICs, tumor-infiltrating immune cells; TIL, tumor-infiltrating lymphocytes; TMB, tumor mutation burden; WES, whole exome sequence.

Furthermore, personalized mRNA vaccines encoding tumor-specific neoantigens used alongside immunotherapy are under investigation. The KEYNOTE-942 trial has demonstrated an improved recurrence-free survival (RFS) rate of 79% compared to 62% at 18 months (HR 0.561) by combining the V940 vaccine with pembrolizumab in a population of patients with resected high-risk melanoma [51]. Additionally, ongoing studies like INTerpath-002 (NCT06077760) are examining the role of the V940 messenger RNA vaccine in conjunction with pembrolizumab in the adjuvant setting for patients with completely resected stage II-IIIB NSCLC. Another trial, YE-NEO-001 (NCT03552718), is investigating a personalized neoepitope vaccine using a yeast vector in a similar adjuvant context. Collectively, these innovative modalities offer a more tailored and potentially more effective approach to cancer therapy, targeting the specific characteristics of individual tumors and potentially triggering a more robust immune response.

### Biomarkers-current and future

**FIGURE 1:**
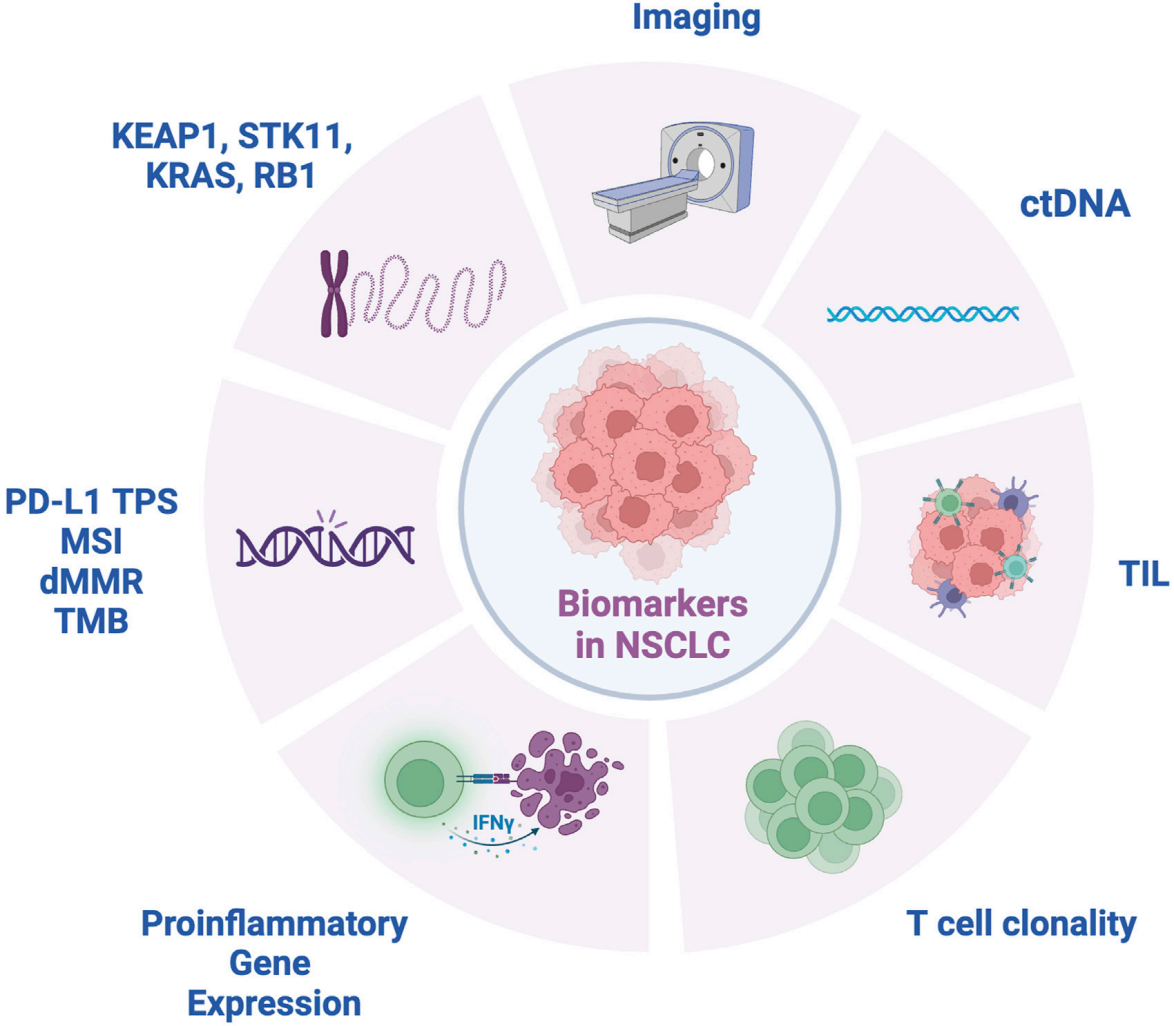
Biomarkers for NSCLC. ctDNA, circulating tumor DNA; dMMR, mismatch repair deficient; IFNγ, interferon-gamma; KEAP1, Kelch-like ECH-associated protein 1; KRAS, Kirsten ras sarcoma oncogene; MSI, microsatellite instability; RB1, retinoblastoma 1 gene; STK11, serine/threonine kinase 11; TIL, tumor infiltrating lymphocytes; TMB, tumor mutation burden; TPS, tumor proportion score.

In the effort to optimize perioperative immunotherapy, biomarker studies aim to identify potential prognostic and predictive correlates of treatment outcomes (Figure 1). For example, integration of circulating tumor DNA (ctDNA) assays has shown promise in perioperative trials. One such application of ctDNA includes monitoring ctDNA dynamics following neoadjuvant chemoimmunotherapy. In operable NSCLC, the NADIM trial showed that pretreatment mutant allele fraction (MAF) < 1% correlated with PFS and OS benefits following neoadjuvant nivolumab and chemotherapy. In addition, clearance of ctDNA after neoadjuvant treatment was associated with improved PFS and OS [10]. CheckMate-816 similarly applied ctDNA dynamics to neoadjuvant chemotherapy, showing higher ctDNA clearance in preoperative nivolumab plus chemotherapy compared to chemotherapy alone. In addition, undetectable ctDNA following neoadjuvant treatment was positively associated with EFS and pCR [8].

**FIGURE 2:**
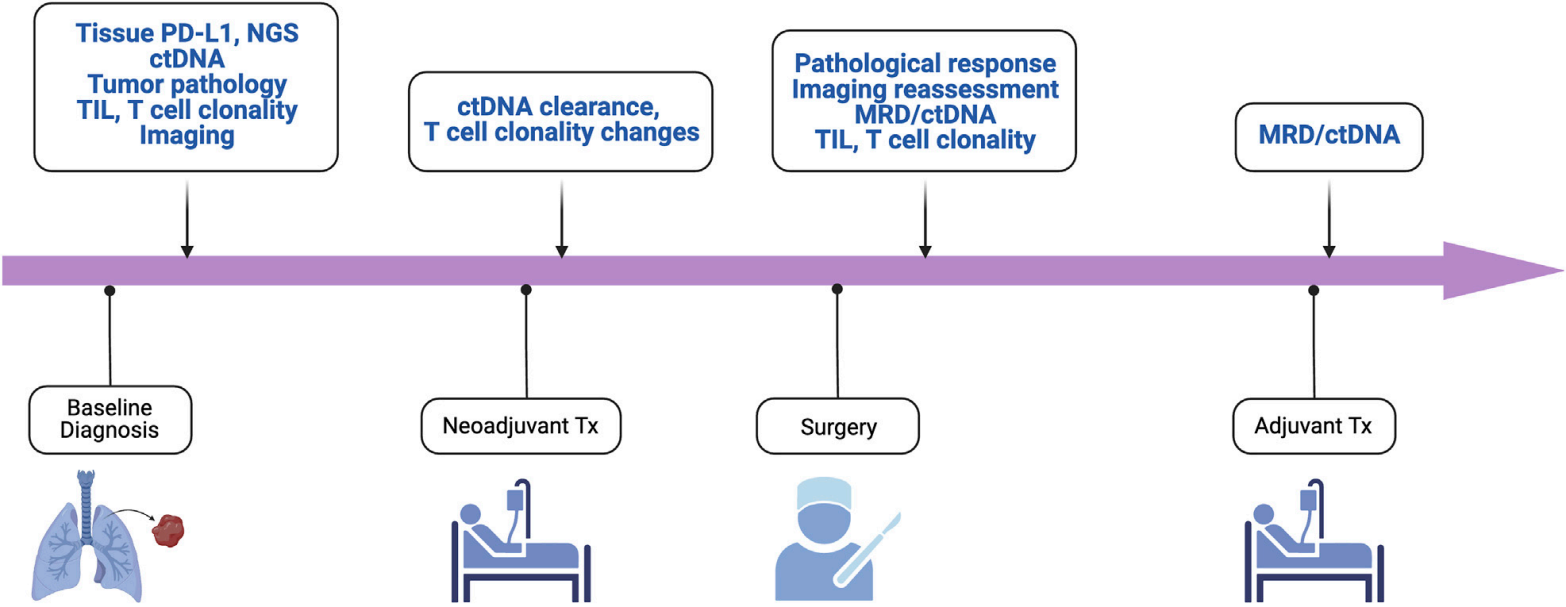
Key biomarkers for enhanced management and trial enrichment. ctDNA, circulating tumor DNA; mPR, major pathological response; MRD, minimal residual disease; NGS, next-generation sequencing; pCR, pathologic complete response; TIL, tumor infiltrating lymphocytes; TMB, tumor mutation burden.

Furthermore, ctDNA has also been applied to the assessment of minimal residual disease (MRD) (Figure 2). Indeed, in early-stage NSCLC treated with surgery and adjuvant chemotherapy and/or radiotherapy, ctDNA detection following resection was associated with clinical recurrence [34]. Similarly, the LUNGCA-1 study has shown a temporal association post-operatively between ctDNA presence and RFS [52]. In the perioperative immunotherapy space, the Impower010 trial showed that ctDNA detection postoperatively was similarly associated with a trend towards worse DFS in both patients treated with adjuvant atezolizumab and best supportive care following adjuvant chemotherapy [53].

Several studies are underway to further elucidate the role of MRD detected via sensitive MRD platforms in perioperative settings. One such trial is NCT04367311, a phase II study using chemoimmunotherapy with atezolizumab looking at ctDNA clearance in MRD-positive patients with resected stage I/IIA NSCLC. ADAPT-E is another phase II study assessing the utility of adjuvant durvalumab for stage I-III NSCLC with ctDNA positivity after definitive surgery or radiation and have completed standard of care chemotherapy as to achieving ctDNA clearance (NCT04585477). Future work using designs similar to the ADAPT-E trial are necessary to investigate whether MRD-positivity using ctDNA can better identify patients at risk of recurrence postoperatively and guide the use of adjuvant immunotherapy to minimize that risk.

Despite the promise that ctDNA may hold as a biomarker of interest, there are important limitations to its use influenced by the inherent sensitivity of ctDNA detection methods [54] and clonal hematopoiesis of indeterminate potential (CHIP) to a lesser extent [55]. For example, ctDNA’s utility is constrained by its limited sensitivity in cases of low tumor burden such as early-stage NSCLC [56] where the sparse release of tumor DNA into the bloodstream may fall below the detection threshold of current technologies. In addition, most trials do not specify CHIP, a condition characterized by the accumulation of somatic mutations in hematopoietic stem cells, which can interfere with the accurate interpretation of ctDNA mutations. This interference is especially problematic when the variant allele frequency (VAF) of ctDNA mutations is low, as DNA shed from white blood cells harboring CHIP mutations may be mistakenly attributed to tumor-derived DNA. Together, these challenges underscore the need for enhanced detection methods and interpretative strategies to accurately discern ctDNA’s true clinical value in the management of cancer patients in neoadjuvant and perioperative contexts.

In metastatic NSCLC, recognized predictive biomarkers for immunotherapy response include PD-L1 tumor proportion score, microsatellite instability (MSI)/deficient mismatch repair (dMMR), and tumor mutational burden (TMB). Based on evidence for these biomarkers in multiple solid tumors, the FDA granted approval for pembrolizumab for MSI-high, dMMR, and TMB-high tumors regardless of tissue type [57]. Nonetheless, in the perioperative immunotherapy space TPS and TMB score have shown inconsistent results. While high PD-L1 TPS was associated with higher mPR rates in the LCMC3 and NEOSTAR studies, later phase trials have not reliably reproduced these findings although certainly general trends are observed of better results in patients with TPS score high positive tumors [58, 59]. In the phase III Checkmate 816 study, both PD-L1 positive and PD-L1 negative patients showed improved pCR rates with neoadjuvant chemoimmunotherapy. Of note, the PD-L1 high patients had a highly impressive close to 50% pCR rate and showed the greatest improvement in EFS(8). In addition, inconsistent results were noted in the adjuvant setting as well where TPS score appeared to correlate with DFS in the Impower 010 study, while the Phase III PEARLS trial found that adjuvant pembrolizumab was associated with longer DFS across all PD-L1 subgroups [60]. As for TMB, its role remains unclear in the perioperative space as several studies thus far including the Checkmate 816, LCMC3, and NADIM trials evaluating TMB and its association with pCR have failed to show a significant relationship [8, 10, 59].

The perioperative setting is ideal for studying novel biomarkers by examination of both pre-treatment and post-treatment tissue obtained following surgical resection. For example, several trials have investigated how the immunophenotype of the tumor microenvironment and in circulating peripheral blood may relate to perioperative immunotherapy outcomes. T-cell repertoire was evaluated in an early-phase trial of neoadjuvant nivolumab showing that tumors demonstrating a mPR showed a higher clonality of the T-cell population in both the tumor and peripheral blood [7]. LCMC3 employed similar methods to evaluate T-cell responses in resected NSCLC following neoadjuvant atezolizumab finding that tumors with mPR were significantly associated with an expansion of peripheral blood-activated CD8^+^ T cells [59]. The NEOSTAR trial studying neoadjuvant nivolumab and ipilimumab versus nivolumab evaluated the immune cell infiltration of pre- and post-therapy tumor specimens using multiplex immunofluorescence and demonstrated that dual-immunotherapy combination induced greater overall tumor infiltration of CD3^+^ and CD3^+^CD8^+^ T lymphocytes, tissue-resident memory cells, and effector memory T cells than single-agent nivolumab [58]. In the NADIM trial investigating neoadjuvant nivolumab plus chemotherapy, tumors achieving pCR were associated with a proinflammatory gene expression profile and higher upregulation of IFN-γ-responsive genes involved in antitumor response [61]. Those tumors without pCR, however, showed upregulation of genes related to proliferation. Additionally, peripheral blood collected from patients enrolled in the NADIM trial showed a differential profile of immune parameters based on pCR or non-pCR, such as higher CD4^+^ PD-1+ cells and lower monocyte CTLA-4 expression in patients with pCR [62]. Future studies are necessary to elucidate how pathologic correlates such as T cell clonality, immune cell infiltration, and immune gene expression in the tumor microenvironment and peripheral blood relate to perioperative immunotherapy response.

Another group of biomarkers of uncertain significance are several key somatic mutations associated with poor response to immunotherapy. For example, a *post hoc* analysis of the POSEIDON trial evaluating combined PD-L1 and CTLA-4 inhibition with durvalumab and tremelimumab plus chemotherapy in metastatic NSCLC showed that patients with KEAP1, STK11, and KRAS mutations benefited more from combination immunotherapy [63]. In addition, results from the perioperative NADIM trial showed that tumors with KEAP1, STK11, and RB1 mutations were less likely to show a benefit from preoperative immunotherapy [10]. Further efforts will be required to determine how the presence such molecular alterations can guide perioperative immunotherapy treatment strategies.

Lastly, radiomic biomarkers have shed light to predicting response to immunotherapy in the perioperative settings. Literature shows imaging biomarkers such as maximum standardized uptake value (SUVmax), metabolic tumor volume (MTV) and total lesion glycolysis (TLG) from positron emission tomography/computed tomography (PET/CT) scans have demonstrated prognostic significance in resectable NSCLC. A meta-analysis found that high SUVmax, MTV, and TLG correlated with lower disease free survival (HR of SUVmax = 2.43, MTV = 2.49, TLG = 2.97) and OS (HR SUVmax = 1.52, MTV = 1.91, TLG = 1.94) in resectable NSCLC [64]. Sun et al also demonstrated high radiomic based biomarker of tumor infiltration with CD8 cells was associated with better overall survival of 24.3 months compared to 11.5 months in those with low radiomic score (HR 0.58) [65]. Zerunian et al assessed CT-derived texture parameters less than 56.2 mean value of positive pixels to be associated with lower OS and PFS with HR 0.89 with pembrolizumab use [66]. Further work is needed to explore how imaging biomarkers can be systematically integrated into perioperative trials to enhance prognostication and therapeutic approaches.

The development of precise biomarkers is critical in the perioperative setting to optimally treat patients with NSCLC. Current limitations may hinder personalized treatment planning and often lead to a trial-and-error approach, potentially delaying the identification of the most effective treatment for individual patients. The development and validation of reliable predictive biomarkers is essential to optimize treatment selection, enhance response rates, and avoid unnecessary toxicity from ineffective therapies.

## Conclusion

In this review, we summarize the current rapidly evolving landscape of perioperative therapy for NSCLC. The evidence gathered from recent clinical trials underscores the potential of neoadjuvant approaches to improve and augment surgical outcomes, enhance pathological response rates, and ultimately, increase overall survival rates for patients with resectable NSCLC. Further challenges in optimizing patient selection to identify ideal candidates for neoadjuvant treatments, duration of treatment, and optimal treatment regimen are still ongoing and need to be supported. Integration of molecular profiling and the development of predictive biomarkers hold promise for personalizing neoadjuvant treatment approaches, potentially enabling the tailoring of therapy to individual patient characteristics and tumor biology. Moreover, the exploration of novel therapeutic agents and combinations, as well as the innovative endpoints in trial designs, will be crucial in overcoming resistance mechanisms and improving patient outcomes.

## Abbreviations

ALK: Anaplastic Lymphoma Kinase; CHIP: Clonal hematopoiesis of indeterminate potential; dMMR: Deficient mismatch repair; EFS: Event free survival; EGFR: Epidermal Growth Factor Receptor; mAb: Monoclonal Antibody; MAF: Mutant allele fraction; mPR: major pathological response; MSI: Microsatellite instability; NSCLC: Non-small cell lung cancer; OS: Overall survival; pCR: Pathologic complete response; PDC: Platinum doublet chemotherapy; TMB: Tumor mutation burden; TME: Tumor microenvironment.
